# Evidence on designing sanitation interventions

**DOI:** 10.1016/j.jdeveco.2024.103316

**Published:** 2024-10

**Authors:** Britta Augsburg, Andrew Foster, Terence Johnson, Molly Lipscomb

**Affiliations:** a Institute of Fiscal Studies, UK; b Brown University, United States; c University of Virginia, United States

## Abstract

Sanitation is a public good, the responsibility for which is shared between households and the government. Interventions in the sector, therefore, must be designed with an eye toward reducing crowd out. We discuss the new findings on sanitation provision from the 12 papers in this special issue in the context of a simple model of household choice of levels of sanitation investment in the face of joint responsibility between the government and households over sanitation. The model provides micro-foundations for understanding when we should be particularly concerned about the potential for crowd-out together with intuition for the implications of the choice of intervention design between information, in-kind transfers, cash transfers, and subsidies. We use the framework of the model to discuss the findings of the papers in this special issue.

## 1 Introduction

Strong externalities based on the local transmission of diseases means households will typically under-invest in sanitation, relying on public sector investment and enforcement to maintain environmental standards. Conversely, the government faces competing priorities for scarce public funds, many of which are higher profile and more attractive than sanitation. Both households and the government would prefer that the other party allocate more resources to the good so that they can focus on other priorities. In this summary article, we discuss how government interventions can be designed to encourage greater household investment in sanitation. We further present the evidence from the articles in this special issue on the effectiveness of various types of sanitation interventions in inducing improvements in coverage and welfare.

A common thread in the existing literature on sanitation is substantial variation in treatment effects, with much of the literature finding null impacts or impacts that are not sustained over time.^1^ Estimates of willingness to pay for sanitation are often disappointingly low. At first sight, this is difficult to reconcile with the potentially large welfare impacts of improving sanitation in terms of reducing child diarrhea rates and improving levels of health and hygiene (Fewtrell et al., 2005). This leads to difficulties in interpreting the impacts for policy makers who need to make decisions about how to invest limited budgets in sanitation inputs versus other policy priorities – the key lesson from the literature often seems to be that the outcome of sanitation policy is highly context dependent.

Particularly because of the prevalence of null results in sanitation interventions, despite the clear health benefits of sanitation, a model can help in elucidating reasons why some interventions may not lead to significant levels of take-up. The model shows that these low estimates may not be due to low demand for sanitation, but to the shared responsibility for sanitation investment between households and the government, and the incentives that shared responsibility creates. We show that the form of government intervention can be very important in determining the household response to the intervention and the overall impact of the intervention.

The papers in this special issue reflect the fact that interventions can have mixed results: substantial welfare impacts are suggested by improvements in sanitation (Deutschmann et al., 2023; Augsburg et al., 2023a), but there is wide variation in the effectiveness of interventions: demand-side interventions increase take-up of toilets (Augsburg et al., 2023b), but average intervention effects can be short-lived and disappear a few years after the intervention (Augsburg et al., 2022). While externalities have received considerable attention to explain low levels of individual investment in sanitation (see, for example, Gautam (2023) or Guiteras et al. (2015)), the observed nonlinearities in the impacts of sanitation interventions suggest that externalities may not fully explain low sanitation provision. Policy design should take into account household trade-offs and the potential for crowd-out.

Where investment in sanitation is observable, the welfare improvements associated with increased government provision can be substantial. Kresch et al. (2023) shows that households with sewerage have higher favorability rankings of the government and are more willing to pay their property tax bills. But the inverse is also true: Allakulov et al. (2023) shows that improved transparency has little effect on the availability of sanitation in schools where access was already low: information may not be enough to change behavior if the marginal benefit of other consumption is high relative to the marginal benefit of sanitation investment.

In the next section, we propose a simple model of household decision-making under joint responsibility for sanitation. The theory has implications for the welfare effects of sanitation and the demand for sanitation at the household level, and provides a framework for thinking about sanitation interventions; both for the papers in this special issue and the findings of other work. The model illustrates how the household response to different forms of government intervention (wealth transfers, information transfers, in-kind transfers and subsidies) differs in terms of crowd out and household choice. We discuss how the papers in this special issue contribute to the understanding of the impact of key interventions in sanitation policy, and the implications for how governments and NGOs might choose to invest in sanitation or design policy in order to optimally use the marginal dollar of spending in the sector.

## 2 Household choice of provision of public services

A representative household has utility *u*(*y*) for consumption of non-sanitation goods and services, *y*. They face non-negativity constraints that consumption *y* and household sanitation investment *s* must both be non-negative, and a standard budget constraint that total expenditure must be less than wealth, *ps* + *y* ≤ *w*, where *p* is the price of sanitation services and *w* is household wealth. For convenience, *u* is strictly concave and strictly increasing, and satisfies lim_*c*→0_*u*′(*c*) = *∞*, so that corner solutions where the household spends all its money on sanitation services are not possible.

The choices made by the household determine the overall quality of the environment, taking as given the level of spending on sanitation that the government has made. The household determines its sanitation spending taking into account the reduction in the probability of illness that would result in lost wages and the avoidance of a potentially painful or fatal health condition. To reflect the impact of spending on the environment, there is a function *π*(*T*) that aggregates total spending *T* = *s* + *g* on sanitation into the proportion of the population that becomes ill or is negatively affected by sanitation-related illness and inconvenience. There is also a cost that accrues to a sick household, *c*_*h*_: this is the perceived cost to the household, and may shift with more information about the actual costs of sanitation-related illness. For simplicity, assume that *π*(*T*) is non-negative, strictly decreasing and convex, so that additional spending always improves the quality of the environment, and does so at an increasing rate. Then the social cost of pollution is −*π*(*T*)(*c*_*h*_), which is decreasing and concave in total spending *T* = *s* + *g*, while the expected cost to a given household is −*π*(*T*)*c*_*h*_. While the definition of these variables in terms of public health and disease risk, they can also reflect more generally all the costs and benefits that accrue to the household through reducing waste.

### 2.1 Household investment choice

The household chooses (*s*) to maximize the sum:

$$\max_{\left(s\right)}u\left(w-ps\right)-\pi \left(s+g\right)\left(c_{h}\right).$$

The household decides to invest nothing in sanitation (putting it at a corner solution) if:

$$u^{\prime }\left(w\right)>-\frac{\pi^{\prime }\left(g\right)c_{h}}{p},$$

and otherwise equates the marginal benefit of consumption to the marginal benefit of abatement:

$$\underset{\text{Marginal benefit of consumption}}{\underbrace{u^{\prime }\left(w-s^{*}\left(w,g,p,c_{h}\right)\right)}}=\underset{\text{Household sanitation cost ratio}}{\underbrace{-\frac{\pi^{\prime }\left(s^{*}\left(w,g,p,c_{h}\right)\right)c_{h}}{p}}}.$$

Whether or not the household selects a corner solution s*(*w*, *g*, *p*, *c*_*h*_) = 0 depends on both its finances and costs, as well as the level of public provision.

**Fig. 1:**
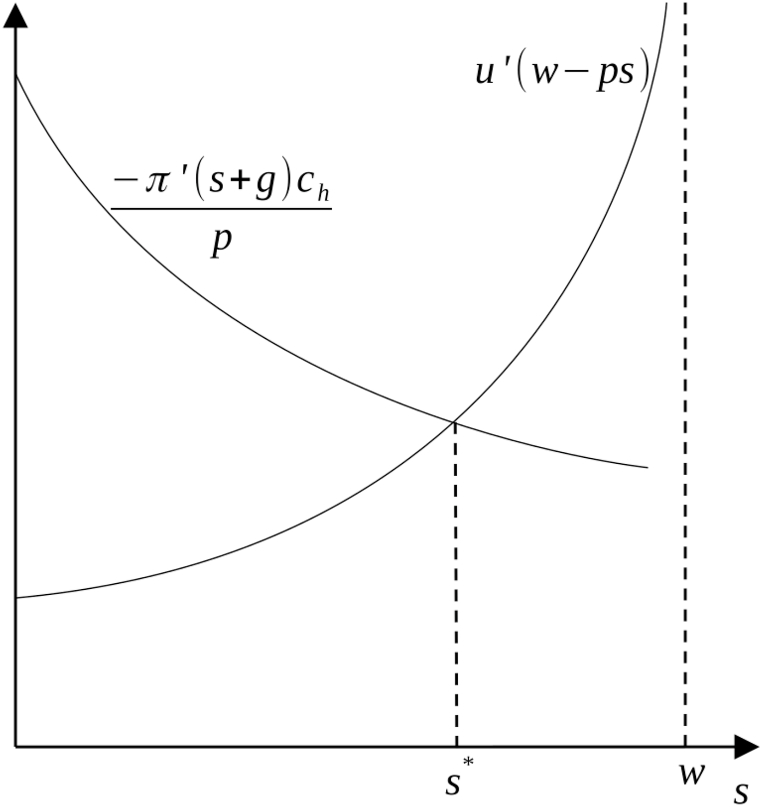
Household sanitation investment determination.

Fig. 1 illustrates in an intuitive way how the household's trade-offs are decided. The household's sanitation cost ratio, −*π*′(g* + *s*)*c*_*h*_/*p*, acts as a declining marginal benefit or demand curve for sanitation, while the household's marginal benefit curve for consumption, *u*′(*w* − *ps*), acts as an increasing marginal cost or supply curve.

### 2.2 Interventions on household wealth, *w*

The model unambiguously predicts that monetary transfers and grants will boost spending on sanitation.^2^

There are, however, several factors that constrain the amount that the household responds:

● The marginal benefit of sanitation goods relative to the marginal benefit of other goods. In some cases, the marginal benefit of other goods may be above sanitation goods, leading to a corner solution of 0 investment in sanitation. Even when wealth is increased, the household will continue to purchase the other goods rather than sanitation goods until the marginal benefit of both goods are equal.
● Because sanitation goods have strong externalities, the marginal benefit to the household of purchasing the goods is lower than the benefit to society, thereby reducing household demand for sanitation goods.

Interventions aimed at improving sanitation availability by increasing household wealth or easing liquidity constraints provide a test of the impact of moving this parameter. Household subsidies have been used extensively in India to increase the uptake of household toilets through the Clean India Campaign.^3^ Augsburg et al. (2023a) show that such policies can lead to general equilibrium effects that are independent of health. In particular, they show that the policy as implemented in India in the late 1990s made marriage more attractive for both sexes and led to a redistribution of gains within the marriage. Deb et al. (2024) evaluate the Clean India Campaign, as implemented in the northern state of Punjab, in combination with a behavioral change campaign and assess the incremental effects of intensive hygiene campaigns in schools and follow-up initiatives in communities. They find that while on average financial incentives are effective in inducing marginal households to invest, open defecation remains present in this population - a fact consistent with other studies that have found that open defecation remains present after the SBM in other Indian states, and indeed, with basically any intervention aimed at tackling open defecation. Even when wealth is increased, households located at a corner solution where the marginal benefit of other goods is above the marginal benefit of sanitation may still not invest in sanitation.

To the extent that households value sanitation but face liquidity constraints that make it difficult for them to invest in toilets, increased access to microfinance could improve toilet coverage. Augsburg et al. (2023b) evaluate the provision of sanitation microcredit to solve the liquidity failure, highlighting how imperfect capital markets can act as an important barrier to sanitation investment for some households. They also provide indirect evidence that price shocks can hinder the conversion of the loan into a functioning toilet, as households living in areas with high toilet construction costs are significantly less likely to convert the loan into a toilet. While easing the liquidity constraint increases demand somewhat, many households divert their sanitation loan to other uses that are more directly valuable to them, thereby limiting potential improvements in local sanitation.

When deciding whether to purchase a toilet, both the actual costs and the perception of costs involved in achieving improved sanitation. Augsburg et al. (2023c) show how the perceptions of costs and benefits of sanitation of *both* husband and wife, and in particular the difference in their expectations, play an important role in initial sanitation investments. They consider two key stages of the investment process and show that the take-up of a sanitation loan is higher in households where the wife has a higher perception of the benefits, while the successful conversion to a toilet depends on differences in cost perceptions, and in particular on the husband's lower cost perception. On average, the husband is at a corner solution where the marginal benefit of other goods is higher than the marginal benefit of sanitation.

### 2.3 Interventions on information and cost of illness or pollution, *c*_*h*_

The model unambiguously predicts that a real or perceived increase in the cost of illness weakly increases household spending.^4^ However, there are a variety of challenges in shifting beliefs.

Where poor sanitation is perceived to have little or no impact on health and welfare, it can be particularly difficult to improve uptake. Attributing health benefits from practices such as handwashing and improved sanitation can be quite difficult, as there are many sources of contamination that may still lead to illness, albeit at a reduced rate. To the extent that households perceive only very limited health benefits from sanitation, they are likely to reduce their investment in sanitation.

Following workshops explaining poor menstrual management as a source of urinary tract infections, Czura et al. (2024) find that there is some increase in the use of the menstrual pads when they are provided for free. However, there are no downstream effects on the women's productivity: the welfare impacts of the interventions are limited to the private benefits to women of improved menstrual practices.

### 2.4 Interventions on government investment or in-kind transfers, *g*

In-kind transfers of sanitation services have a very different impact from transfers of funds or wealth. Cash and in-kind transfers are distinct shocks to the household's decision problem: one reduces the marginal benefit of consumption, while the other affects the marginal benefit of sanitation services. The comparative statics above show that, unlike cash transfers, the impact of in-kind transfers on the equilibrium level of the good can be ambiguous.^5^

Direct government spending on sanitation increases the level of sanitation provision, but also causes crowd-out. Whenever the marginal benefit of abatement is non-decreasing and *π*(*T*) is convex, there will be crowd-out of household investment by government spending. Therefore, increased government spending on sanitation may actually lead to muted responses in terms of increased sanitation as households respond by resetting their own spending priorities.

Crowd out and externalities may have similar impacts. Because the marginal benefit of sanitation improvements is not fully internalized by the household investing in them, the household will under-invest in sanitation. This is compounded by the fact that the government will also invest in sanitation, reducing the marginal benefit of sanitation investments by households. Gautam (2023) integrates sanitation externalities into a model of household choice and estimates the welfare effects of policy interventions, showing that the costs of subsidising sanitation uptake are outweighed by the substantial welfare gains from increased investment in sanitation. Her structural model allows her to calculate price and income elasticities, and by combining these with the interdependence of household decisions within the neighbourhood, she shows that a price subsidy is a significantly more effective policy than unconditional cash transfers, which can be used to make alternative investments from which the recipient may perceive a larger direct benefit.

### 2.5 Interventions directly on price *p* through subsidies

Given the above model, interventions on *g* necessarily lead to crowd out. Of course, this does not mean that the government should cut spending on sanitation, but it does make two very clear predictions: First, that the impact of additional spending will typically be blunted by endogenous reduction in household spending, and, second, that additional spending will never induce households to increase their investment from nothing to something, since they are already at a corner solution.

An alternative intervention would be to provide a subsidy at the household level, reducing the price faced by the household. This indeed induces any household at an interior solution to increase its spending on sanitation,^6^ and can induce households at a corner solution to initiate spending.

But a subsidy policy has its own opportunity cost in the form of spending on other government investment. Intuitively, if the household response to a price reduction and the relative cost of pollution are jointly large enough relative to the marginal benefit of government spending, the government can engage in a kind of “arbitrage”, where it is actually cheaper to reduce the price inducing the household to spend on sanitation rather than to purchase additional sanitation services itself.

One concern with subsidies is that even if the initial purchase is subsidized, ongoing maintenance is often not, and so a household that decides to make the initial purchase as a result of the subsidy may not pay for ongoing maintenance. Bakhtiar et al. (2023) finds relatively large impacts of financial rewards for household sanitation purchases (similar to subsidies), but these impacts are not sustained – they show larger sustained effects for public commitments to purchase sanitation goods. Similarly, Augsburg et al. (2022) shows that sanitation improvements from a CLTS program in Pakistan were on average not sustained over a two year period.

## 3 Conclusion

While many of the impacts of sanitation interventions found in the literature tend to be disappointing, this may not be a simple result of low household demand. Our model suggests a different story – the shared responsibility for sanitation between governments and households leads to underinvestment and crowd-out. The model and the papers in this special issue have five implications for determining optimal sanitation policy:

● If marginal utility *u*′(*y*) is large relative to the weighted marginal benefit from sanitation services, the household can end up at a corner solution, where it spends 0 on sanitation.
● Interventions designed to increase information tend to have limited impacts: households may already have the information, or in cases in which they do not, it may still not alter their sanitation investment decisions.
● Crowd out can be significant: in settings where a program attempts to increase provision through in-kind transfers of sanitation goods, households may respond by reducing their own investment in the sanitation good.
● Households are likely to spend at most a portion of any wealth transfer on sanitation.
● Subsidies may be more cost-effective than in-kind and cash transfers, but maintenance after the programs have ended can be an issue.

Achieving the 6th Sustainable Development Goal on sanitation will require a “big push” on sanitation, as many countries are significantly off target. It is possible that the marginal benefit of sanitation interventions will increase rather than decrease following an increase in sanitation provision, particularly when starting from very low levels of sanitation provision. This would create non-linearities in the health impact of interventions. For example, Cameron et al. (2022) shows that incremental changes in sanitation below 40% coverage have minimal to no impact on overall health levels, but that above 40% coverage children's health improves substantially. Increasing marginal benefits of sanitation provision suggests that such a “big push” by the government or public sector to clean up the environment may be exactly what is required to incentivize households to start investing themselves. Supply-side interventions similar to Deutschmann et al. (2023) provide suggestive evidence that a “big push” on the supply side can have substantial and lasting impacts. Further research is needed to better understand potential non-linearities associated with the benefits of sanitation.

The literature on sanitation impacts, particularly the articles in this special issue, supports these mechanisms as important in limiting the welfare impacts of sanitation interventions. A better understanding of how these mechanisms interact offers the potential to improve policy by directly addressing each of them.

**Table:** 

Paper	Welfare	Intervention				
		**Information**	**Wealth**	**In-kind transfers**	**Price subsidies**	**Supply side**
Augsburg et al. (2023a)	Improved sanitation increases gains from being married, but gains are not evenly distributed.					
Cameron et al. (2022)	Threshold effects of sanitation: child health increases 0.3 std deviations when coverage is 50–75%.	Increase in private sanitation coverage of 7–39 ppts from CLTS.				
Czura et al. (2024)	Improvement of about 6 ppts in propensity to contract UTIs, no impact on productivity from availability of menstrual products.	Workshops improve menstrual health knowledge by 6 ppts with some small accompanying improvements in practices.	Workers provided with free pads are 17.2 ppts more likely to use pads.			
Gautam (2023)	Price subsidies have a regressive effect relative to targeted cash transfers.		Targeted cash transfers increase take-up of sanitation by only 0.2 ppts.		Untargeted price subsidies increase take up by 2.1 ppts.	
Deutschmann et al. (2023)	A 22 ppts reduction in child diarrhea from privatization of treatment centers.					Privatization of treatment centers leads to 74 ppts increase in use of the centers.
Augsburg et al. (2022)		Continued engagement improves sustainability of sanitation by 14 ppts where initial conditions are unfavorable.				
Allakulov et al. (2023)		No impact of scorecards and workshops on school sanitation provision.				
Bakhtiar et al. (2023)		Public commitment to purchase latrines induces an enduring 4.2–6.3 ppts increase in latrine ownership.			Financial rewards for latrine ownership increase ownership by 7.5 ppts in short term, but no effect in medium term.	
Augsburg et al. (2023b)			Labeled microcredit increases sanitation ownership by 9 ppts.	Unanticipated delays to receiving government sanitation subsidies and high toilet construction costs impede conversion of sanitation loans to sanitation investments.		
Augsburg et al. (2023c)			Uptake of sanitation loans is 23–27 ppts higher among households where the wife has higher benefit perception; loans are used to construct toilets only when husbands have higher cost perceptions and women are involved in decision-making. For this groups, toilet uptake increases by 16 ppts.			
Kresch et al. (2023)	Improvements to sewerage increase propensity to pay taxes by 15 ppts and approval of government by 4 ppts (10 ppts for tax compliant households).					
Deb et al. (2024)		Households treated with information treatments as well as subsidies for toilet construction were more likely to have increased access to toilets at endline.			Access to toilets increased by 6.1–11.1 percentage points and ownership of toilets increased by 5.7–10.7 percentage points following a subsidy for toilet construction in households without toilets.	

## Footnotes

1 Three large randomized controlled trials (RCTs) of WASH interventions (known as the WASH-Benefits Bangladesh, WASH-Benefits Kenya, and the Sanitation Hygiene Infant Nutrition Efficacy (SHINE) trials) were unprecedented in scale and cost, but showed no impact on child stunting, and a reduction in diarrhea only in only one of the three settings, Bangladesh. A consensus paper (Cumming et al., 2019; Null et al., 2018; Humphrey et al., 2019) on these studies discusses how different settings may require different WASH interventions, and how the same interventions may even have different health impacts in the same settings at different times.

2 The comparative static of *s** with respect to *w* is $\frac{\partial s^{*}\left(w,g,p,c_{h}\right)}{\partial w}=\frac{-pu^{\prime \prime }\left(w-ps^{*}\right)}{-\left[p^{2}u^{\prime \prime }\left(w-ps^{*}\right)-\pi^{\prime \prime }\left(s^{*}+g\right)c_{h}\right]}>0.$.

3 The current policy is called the Swatch Bharat Mission, or Clean India Campaign, which followed the Total Sanitation Campaign (TSC).

4 The comparative static of *s** with respect to *c*_*h*_ is $\frac{\partial s^{*}\left(w,g,p,c_{h}\right)}{\partial c_{h}}=\frac{-\pi^{\prime }\left(s^{*}+g\right)}{-\left[p^{2}u^{\prime \prime }\left(w-ps^{*}\right)-\pi^{\prime \prime }\left(s^{*}+g\right)c_{h}\right]}>0$.

5 The comparative static of *s** with respect to *g* when *π*(*T*) is convex is: $\frac{\partial s^{*}\left(w,g,p,c_{h}\right)}{\partial g}=\frac{-\rho \pi^{\prime \prime }\left(s+g\right)c_{h}}{-\left[u^{\prime \prime }\left(w-s^{*}\right)-\rho \pi^{\prime \prime }\left(s^{*}+g\right)c_{h}\right]}<0,$.

6 The comparative static of household spending on *p* is $\frac{\partial s^{*}\left(w,g,p,c_{h}\right)}{\partial p}=\frac{-u^{\prime }\left(w-ps\right)+p^{2}u^{\prime \prime }\left(w-ps\right)}{-\left[p^{2}u^{\prime \prime }\left(w-ps^{*}\right)-\pi^{\prime \prime }\left(s^{*}+g\right)c_{h}\right]}<0,$ so that a reduction in *p* increases household sanitation spending.
